# Cytological identification of *Blastocystis hominis* in the ascites of a patient with rectal carcinoma: a case report

**DOI:** 10.3389/fmed.2025.1539661

**Published:** 2025-03-04

**Authors:** Qian Lin, Jun Huang, Yuanyuan Chen, Xiaoli Wu, Yuhua Ma, Qing Yang, Pan Long, Xin Chen

**Affiliations:** ^1^Department of Laboratory Medicine, Affiliated Hospital of Southwest Jiaotong University, The Third People’s Hospital of Chengdu, Chengdu, Sichuan, China; ^2^Department of Ophthalmology, General Hospital of Western Theater Command, Chengdu, China

**Keywords:** *Blastocystis hominis*, cytology, intestine perforation, colorectal carcinoma, case report

## Abstract

**Introduction:**

*Blastocystis hominis* (*B. hominis*), a protozoan parasite often found in the human intestinal tract, is typically identified through fecal examination. Its presence in ascitic fluid is quite uncommon, making such a detection significant in the context of medical diagnosis.

**Case presentation:**

In this case report, we describe a 53-year-old female patient who presented with a 6-month history of recurrent diarrhea and fecal incontinence subsequent to the diagnosis of rectal signet ring cell carcinoma. The patient was discovered to have a severe abdominal infection, with *B. hominis* identified in both the abdominal cavity and the intestinal tract. Marked improvement in diarrheal symptoms was achieved following peritoneal lavage with metronidazole.

**Conclusion:**

This case underscores the significance of detecting *B. hominis* infection in the ascitic fluid of a patient afflicted with rectal signet ring cell carcinoma. *B. hominis*, a prevalent opportunistic pathogen, often exploits the compromised immune states and nutritional deficiencies prevalent in cancer patients, rendering them more susceptible to such infections. It is imperative to enhance diagnostic accuracy and mitigate the risk of misdiagnosis to subsequently improve the clinical outcomes and overall quality of life for individuals battling cancer.

## Background

*Blastocystis hominis* is a ubiquitous protozoan that inhabits the human large intestine. Its prevalence ranges approximately 10% in developed countries, yet this figure escalates to between 50 and 60% in developing nations (1). The pathogenicity of *B. hominis* remains a subject of debate, as the majority of individuals carrying this bacterium exhibit no discernible clinical symptoms. Nevertheless, there are exceptions, particularly among patients who have concurrent infections or compromised immune systems. These individuals may experience a range of symptoms including abdominal pain, diarrhea, nausea, and vomiting, with severe cases potentially leading to fatal outcomes (2–5). In this report, we delve into the uncommon detection of *B. hominis* in the abdominal fluid of a patient diagnosed with intestinal adenocarcinoma. Our findings shed light on the complexities of managing *B. hominis* infections in the context of patients grappling with malignant tumors, offering valuable insights for clinical practice.

## Clinical case presentation

A 53-year-old female patient was admitted to the Gastroenterology Department at The Third People’s Hospital of Chengdu, presenting with a six-month history of recurrent diarrhea and a one-month history of abdominal bloating. She had no prior medical record of diabetes, hypertension, coronary artery disease, hepatitis B infection, or tuberculosis. Additionally, the patient denied any history of drug or food allergies, blood transfusions, surgical procedures, or injuries.

**Figure 1:**
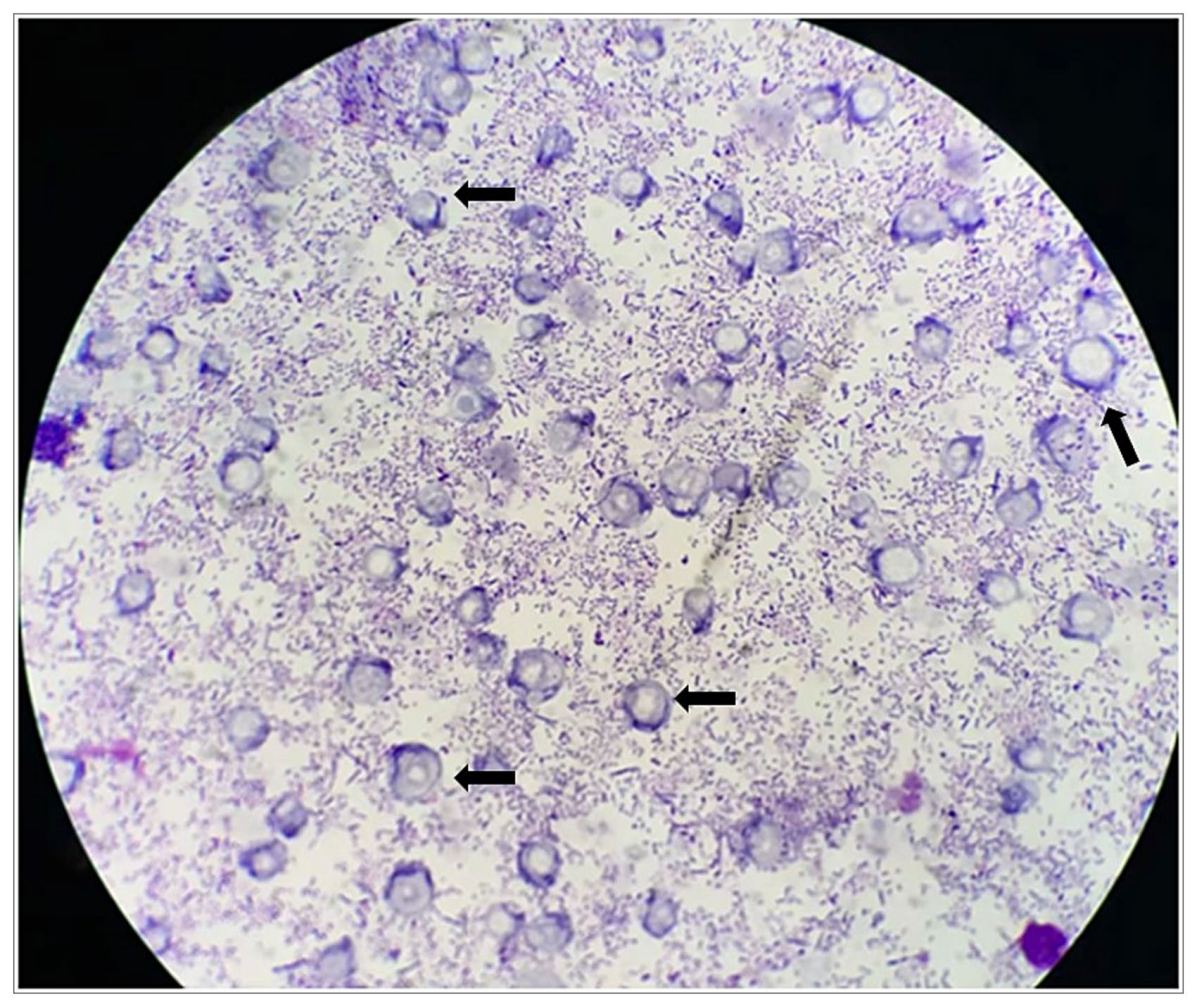
*Blastocystis hominis* appears in ascites.

**Figure 2:**
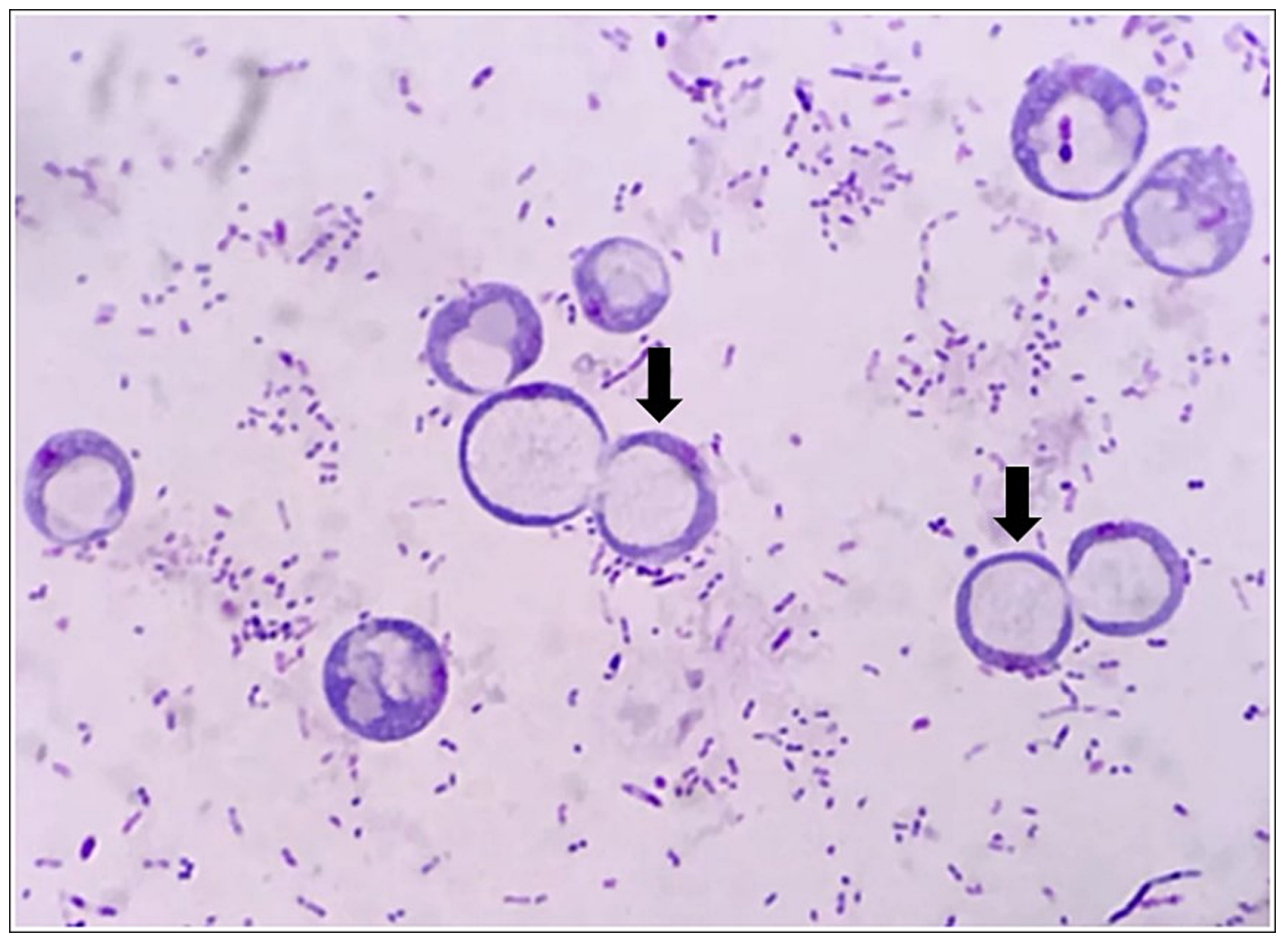
*Blastocystis hominis* appears in ascites (zoom in).

**Figure 3:**
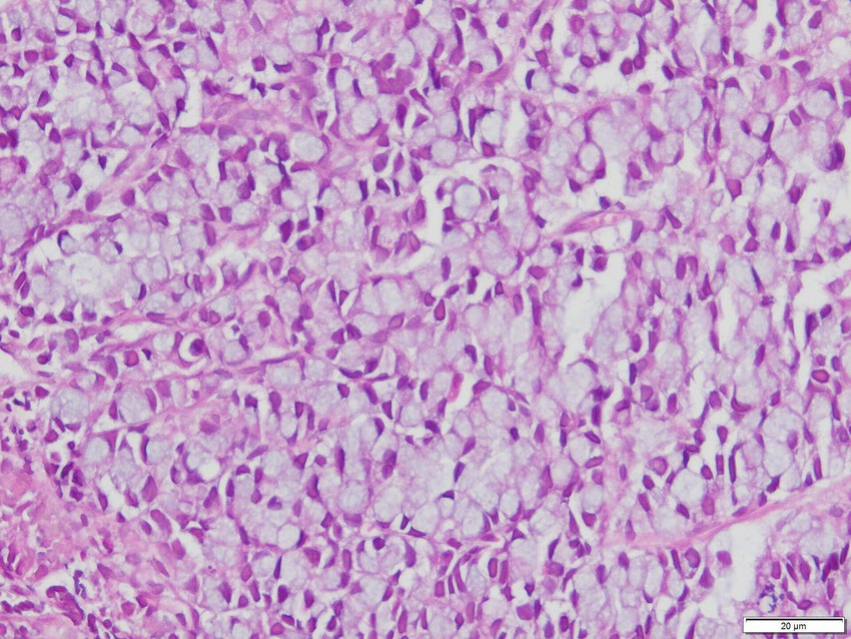
H&E stained microscopic view of signet ring cell carcinoma biopsy (100x Magnification).

**Figure 4:**
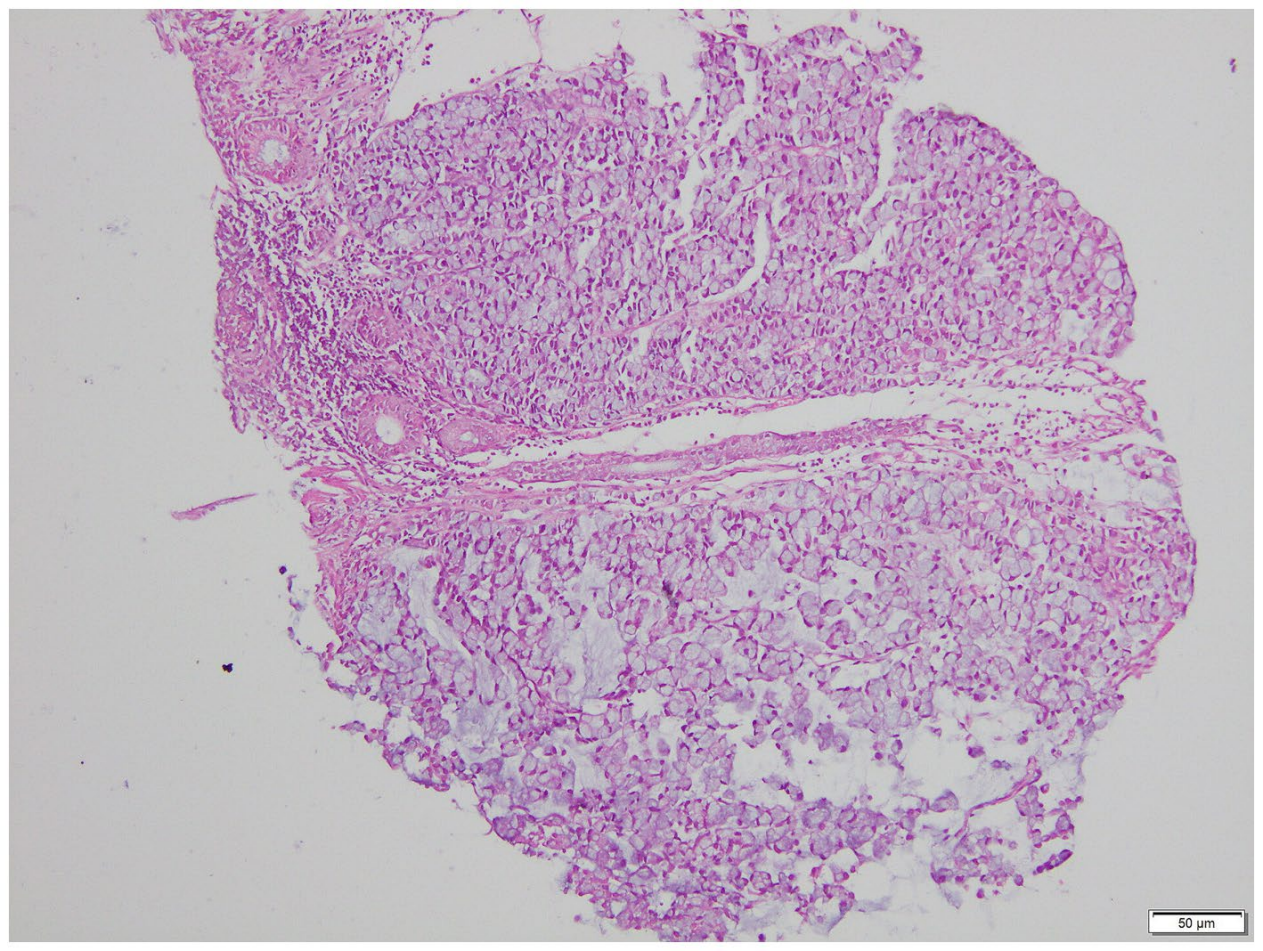
H&E stained microscopic view of signet ring cell carcinoma biopsy (400x Magnification).

**Figure 5:**
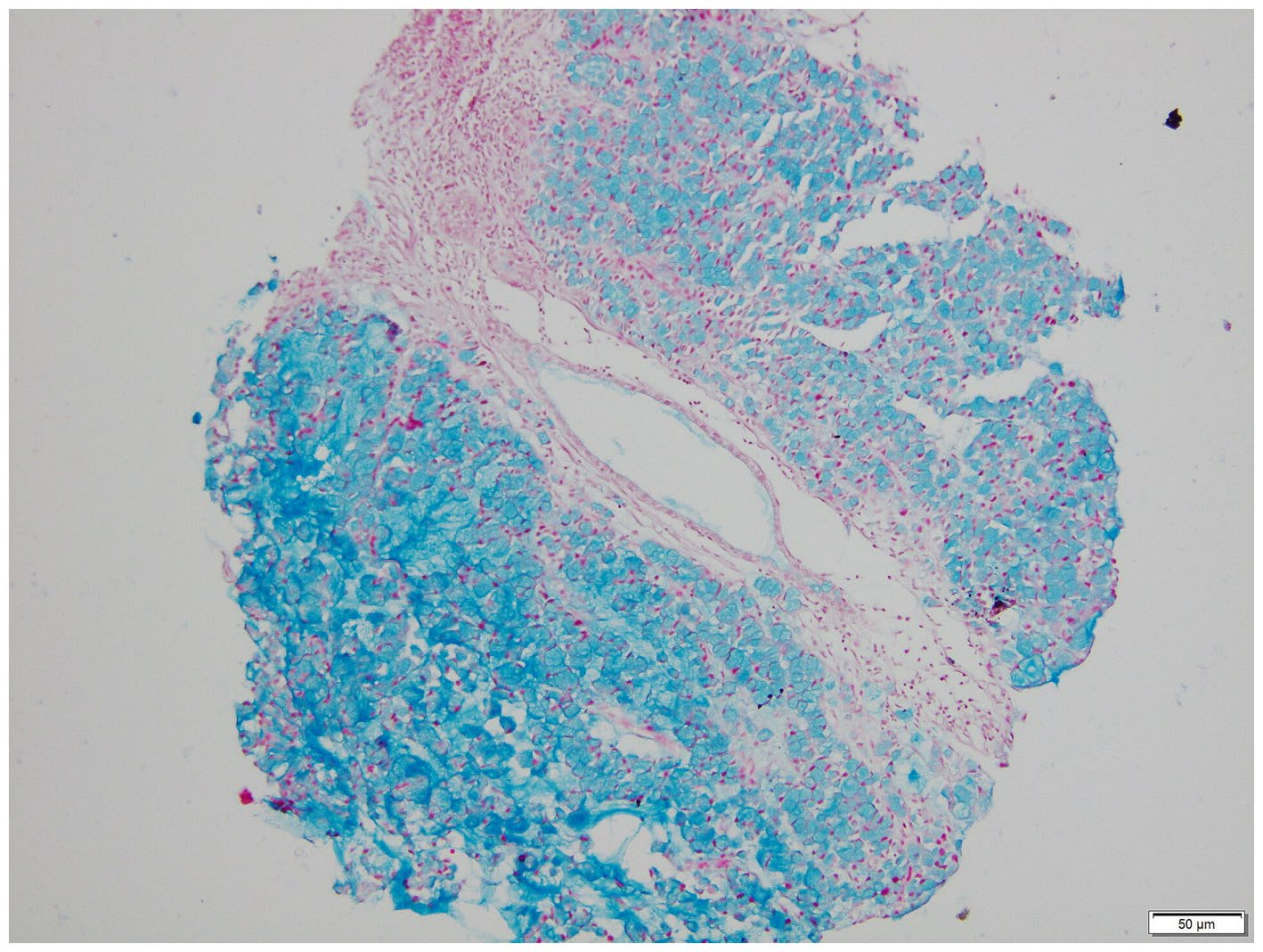
AB-PAS stained microscopic view of signet ring cell carcinoma biopsy (100x Magnification).

The patient presented with abdominal distention and a positive shifting dullness sign upon physical examination. A computed tomography (CT) scan of the abdomen and pelvis revealed massive ascites, enlarged lymph nodes in the retroperitoneum, abdomen, and pelvis, and a suspicious nodule on the right hemidiaphragm, indicative of potential metastasis. Cytological analysis of the ascitic fluid showed a high concentration of nucleated cells, bacteria, and ruptured cells (Table 1). Wright’s stain identified round, lightly stained vacuole protozoa of varying sizes with large transparent bodies, consistent with *B. hominis* (as indicated by arrows in Figures 1, 2). We then conducted a routine and microscopic examination of the patient’s stool samples, which also uncovered the presence of Blastocystis (Table 2). Ascitic fluid culture further revealed an infection with *Escherichia coli* (*E. coli*), and confirmed that the isolate *E. coli* was positive for Extended-Spectrum Beta-Lactamases (ESBLs). Susceptibility testing further showed that the ESBL-producing *E. coli* was sensitive to meropenem. The enteroscope revealed lesions encircling the intestine, accompanied by erosive, congestive, edematous, and hardened mucosa. A subsequent biopsy of the mass identified it as a signet ring cell carcinoma (Figures 3–5). Genetic testing of the rectal biopsy specimen ruled out K-ras gene mutation. Based on these findings, the patient was diagnosed with severe abdominal infection (*B. hominis* and*E. coli*), rectal signet ring cell carcinoma with metastasis to the retroperitoneal lymph nodes, peritoneum, and diaphragm, severe abdominal infection, *B. hominis* infection affecting the abdomen and intestine, severe malnutrition, acute enteritis, and hypoalbuminemia, carcinomatous ascites.

**Table 1:** The laboratory test.

Parameters	Results	Reference range
White blood cell count (per uL)	6,840	4,000-10,000
Neutrophils %	81.2	50–70
Hemoglobin (g/dL)	9.5	11–15
Platelet (per uL)	76,000	100,000-300,000
C-reactive protein (mg/L)	225.7	0–5.0
Total bilirubin (umol/L)	29.89	<17.1
Albumin (g/dL)	2.3	4–5.5
Aspartate aminotransferase (IU/L)	15.6	0–40
Alanine aminotransferase (IU/L)	10.8	0–40
Total cells in ascites (per uL)	15,680	/
Nucleated cells in ascites (per uL)	13,440	/

**Table 2:** The stool routine examination.

Item		Result	Reference range
Stool routine	Color	Brownish	Brownish
	Consistency	Loose stools	Formed soft stools
	Red blood cells	Positive 1+	Negative
	White blood cells	Positive 1+	Occasional
	Pus cells	Negative	Negative
	Phagocytosis cells	Negative	Negative
	Fungi	Negative	Negative
	Fat globules	Negative	Negative
Microscopic examination	Protozoa	*Blastocystis hominis* found	Negative
	Ova	Negative	Negative
Occult blood test	Occult blood test	Positive	Negative

The patient’s ascites was effectively managed through paracentesis and subsequent drainage. A comprehensive treatment regimen was implemented, consisting of a combined chemotherapy approach with raltitrexed and tegafur gimeraciloteracil, alongside radiation therapy that incorporated planning target volume techniques. Additionally, cetuximab was administered as part of the targeted therapy. Following peritoneal lavage with metronidazole and levofloxacin, along with the intravenous administration of meropenem, there was a significant reduction in drainage volume, and the ascites odor was considerably diminished. The patient experienced notable improvements in symptoms such as abdominal bloating, pain, and diarrhea. Regrettably, the patient, after a period of hospitalization, was discharged with a palliative care plan and the patient passed away 9 days following their discharge.

## Discussion

*Blastocystis hominis* is a prevalent intestinal protozoan with a global distribution. Despite its widespread presence, the impact of *B. hominis* on human health and its role in disease pathology remain largely enigmatic (2, 6). Several studies have shed light on factors that may contribute to the pathogenicity of *B. hominis* (7–11) with a particular emphasis on the association between the parasite and immunodeficiency or immunosuppression. Notably, *B. hominis* infection has been identified as a significant cause of diarrhea in individuals living with HIV/AIDS, indicating its potential as a pathogenic agent in this vulnerable population (12, 13). Our case report highlights a severe ascites infection caused by *B. hominis* in a patient with signet ring cell carcinoma. This observation suggests a close link between cancer-related immunosuppression and *B. hominis* infection. Consistent with this hypothesis, *B. hominis* infections have been documented in other cancer patients experiencing immune compromise (14). This is further supported by the identification of *B. hominis* in patients with various cancers, including colorectal cancer, haematological malignancies, bladder, breast, lung, pancreas, basal cell carcinoma, laryngeal, renal cell carcinoma, and prostate cancer (15–19).

The detection of *B. hominis* in ascitic fluid, as observed in our patient, could potentially be traced back to the compromised intestinal mucosal barrier resulting from signet ring cell carcinoma and potential microperforations induced by tumor invasion. This compromise enables *B. hominis* to migrate from the intestinal lumen into the abdominal cavity. The observed erosion, congestion, edema, and induration of the intestinal mucosa during enteroscopy further suggest a weakened barrier, facilitating this migration. The pervasive occurrence of *B. hominis* among many cancer types indicates its possible function as an opportunistic pathogen, taking advantage of the impaired immune systems and overall deteriorated health of cancer patients. These findings underscore the propensity of *B. hominis* to cause opportunistic infections. It is worth noting that the infection rates of *B. hominis* are significantly higher in patients with digestive system tumors, especially colorectal cancer, as compared to those with tumors in other bodily systems (18, 20). Furthermore, according to *in vitro* studies (21), *B. hominis* has the ability to stimulate the growth of colorectal cancer cell lines by suppressing apoptosis. Additional studies (22, 23) have revealed that *B. hominis* can also enhance cancer cell proliferation by downregulating the host’s immune response, indicating a potential involvement of *B. hominis* in the development of colorectal cancer. The rapid proliferation of *B. hominis* in immunocompromised hosts can lead to a spectrum of intestinal pathologies, ranging from mild to severe. It is imperative for medical professionals to recognize that anti-tumor treatments in cancer patients may further suppress the immune system, potentially triggering the reactivation of dormant *B. hominis* or new infections (8). Our study serves as a stark reminder of the critical importance of timely diagnosis of *B. hominis* infections for the appropriate management and treatment of cancer patients.

In the majority of clinical laboratories, microscopic examination of stained slides remains the gold standard for diagnosing *B. hominis* infections. Drawing from personal experience, several strategies can enhance the detection rate of this protozoan. Firstly, conducting multiple stool examinations can significantly boost the diagnostic yield, particularly for patients presenting with chronic diarrhea. Our laboratory protocol involves the use of normal saline to prepare direct smears of stool samples. These smears are initially scanned at low magnification for objects resembling fat globules, starch granules, or white blood cells. Upon identifying suspicious structures, a coverslip is applied, and the slide is then examined at high magnification. *B. hominis* typically appears colorless or light yellow, with a round or oval shape, varying in size. It features a large, transparent body encircled by a thin rim of cytoplasm, within which a few refractile bodies may be observed. The images presented in this article depict vacuolar protozoa with thick walls after staining, showcasing a centrally located, darkly stained area that pushes the nucleus (appearing purple) to the periphery of the organism. In addition to direct smears with normal saline, various staining techniques can be employed to improve detection, including iodine staining, Giemsa staining, iron hematoxylin staining, and direct culture methods. Lastly, for ascites samples, a routine procedure involves centrifugation at 1500 revolutions per minute for 5 min. The sediment at the bottom is then used to create a thin, uniform film. After drying, Wright’s staining is applied, which has been shown to markedly enhance the detection rate of *B. hominis*. Through above described methods, including direct fecal smear examination and staining techniques, this study achieved successful identification of *B. hominis*. Although these methods are simple and rapid, they possess certain limitations, such as insufficient sensitivity. This limitation primarily stems from the polymorphic characteristics, size variations, and varying cell counts of the parasite, which complicate microscopic detection. In particular, a low cell count within the species may have resulted in a higher incidence of false negative results. Compared to these methods, molecular biological diagnostic techniques, represented by PCR technology, offer a sensitive, specific, and reliable method for detecting *B. hominis*. Additionally, these techniques allow for the identification of genetic subtypes of *B. hominis*, providing a valuable foundation for potential correlational research between the epidemiology of *B. hominis*, its genotype, and disease patterns.

Our study underscores the detrimental impact of *B. hominis* infection in cancer patients by identifying its presence in ascites samples. The current research elucidates that significant strides remain to be made in understanding the pathogenicity of *B. hominis*, developing effective treatment strategies, and implementing robust control measures to mitigate its harmful effects.

## Data Availability

The raw data supporting the conclusions of this article will be made available by the authors, without undue reservation.
